# ECM Stiffness-Induced Redox Signaling Enhances Stearoyl Gemcitabine Efficacy in Pancreatic Cancer

**DOI:** 10.3390/cancers17050870

**Published:** 2025-03-03

**Authors:** Shuqing Zhao, Edward Agyare, Xueyou Zhu, Jose Trevino, Sherise Rogers, Enrique Velazquez-Villarreal, Jason Brant, Payam Eliahoo, Jonathan Barajas, Ba Xuan Hoang, Bo Han

**Affiliations:** 1 Department of Surgery, University of Southern California, Los Angeles, CA 90089, USA; shuqingz@usc.edu (S.Z.); jb35313@usc.edu (J.B.); baxuanho@usc.edu (B.X.H.); 2 College of Pharmaceutical Science, Florida A&M University, Tallahassee, FL 32307, USA; edward.agyare@famu.edu (E.A.); xue.zhu@famu.edu (X.Z.); 3 Division of Surgical Oncology, School of Medicine and Surgeon, Virginia Commonwealth University, Richmond, VA 23284, USA; jose.trevino@vcuhealth.org; 4 Departments of Biostatistics, College of Public Health and Health Professions, UF Health Cancer Center, University of Florida, Gainesville, FL 32611, USA; sherise.rogers@medicine.ufl.edu (S.R.); jobrant@ufl.edu (J.B.); 5 Department of Integrative Translational Sciences, City of Hope, Duarte, CA 91010, USA; evelazquezvilla@coh.org; 6 Department of Biomedical Engineering, University of Southern California, Los Angeles, CA 90007, USA; peliahoo@radiosoninc.com

**Keywords:** PDAC, patient-derived organoids, matrix stiffness, drug resistance, stearoyl gemcitabine, oxidative stress

## Abstract

Background: Pancreatic ductal adenocarcinoma (PDAC) remains one of the most lethal malignancies, largely due to its dense fibrotic stroma that promotes drug resistance and tumor progression. While patient-derived organoids (PDOs) have emerged as promising tools for modeling PDAC and evaluating therapeutic responses, the current PDO models grown in soft matrices fail to replicate the tumor’s stiff extracellular matrix (ECM), limiting their predictive value for advanced disease. Methods: We developed a biomimetic model using gelatin-based matrices of varying stiffness, achieved through modulated transglutaminase crosslinking rates, to better simulate the desmoplastic PDAC microenvironment. Using this platform, we investigated organoid morphology, proliferation, and chemoresistance to gemcitabine (Gem) and its lipophilic derivative, 4-N-stearoyl gemcitabine (Gem-S). Mechanistic studies focused on the interplay between ECM stiffness, hypoxia-inducible factor (HIF) expression, and the nuclear factor erythroid 2-related factor 2 (Nrf2) pathway in drug resistance. Results: PDAC organoids in stiffer matrices demonstrated enhanced stemness features, including rounded morphology and elevated cancer stem cell (CSC) marker expression. Matrix stiffness-induced gemcitabine resistance correlated with the upregulation of ABC transporters and oxidative stress adaptive responses. While gemcitabine activated Nrf2 expression, promoting oxidative stress mitigation, Gem-S suppressed Nrf2 levels and induced oxidative stress, leading to increased reactive oxygen species (ROS) and enhanced cell death. Both compounds reduced HIF expression, with gemcitabine showing greater efficacy. Conclusions: Our study reveals ECM stiffness as a critical mediator of PDAC chemoresistance through the promotion of stemness and modulation of Nrf2 and HIF pathways. Gem-S demonstrates promise in overcoming gemcitabine resistance by disrupting Nrf2-mediated adaptive responses and inducing oxidative stress. These findings underscore the importance of biomechanically accurate tumor models and suggest that dual targeting of mechanical and oxidative stress pathways may improve PDAC treatment outcomes.

## Simple Summary

Pancreatic ductal adenocarcinoma (PDAC) is highly lethal, in part due to its stiff, fibrotic tumor environment, which fosters drug resistance. This study developed an organoid model using gelatin-based matrices with varying stiffness to mimic PDAC’s rigid extracellular matrix. Organoids in stiffer matrices exhibited enhanced cancer stem cell traits and greater resistance to gemcitabine (Gem), linked to oxidative stress defenses and drug transporters. A gemcitabine analog conjugated to steric acid (Gem-S) countered these defenses by suppressing Nrf2, a stress response protein, and inducing oxidative stress, leading to higher cancer cell death. Both drugs also reduced hypoxia-inducible factor (HIF) expression, though Gem was more effective. This work highlights the role of tumor stiffness in drug resistance and suggests that targeting mechanical and oxidative stress pathways may enhance PDAC treatment.

## 1. Introduction

Pancreatic ductal adenocarcinoma (PDAC) remains one of the most lethal malignancies, with a dismal five-year survival rate below 11% [1,2]. This poor prognosis stems largely from late-stage diagnosis, aggressive metastasis, and limited therapeutic efficacy [3]. A defining hallmark of PDAC is its dense fibrotic stroma, orchestrated by pancreatic stellate cells (PSCs), which creates a complex and protective tumor microenvironment (TME) [4,5].

While gemcitabine has served as the cornerstone of PDAC chemotherapy for decades, intrinsic and acquired resistance severely limits its clinical impact [6]. Traditional explanations for treatment failure have focused on the physical barrier theory, where dense extracellular matrix (ECM) components impede drug penetration and elevated interstitial pressure restricts drug delivery [7,8]. However, emerging evidence suggests a more nuanced role of the stroma in therapeutic resistance [9,10].

Research has identified several molecular pathways involved in resistance, including altered drug transporter expression, metabolic reprogramming, and enhanced DNA repair mechanisms. Additionally, the dynamic tumor microenvironment (TME) plays a critical role in conferring drug resistance [11].

Recent studies have revealed that stromal mechanics actively reprogram cancer cell behavior beyond simple drug obstruction [10,12]. The dense ECM environment triggers mechanotransduction pathways that enhance invasiveness, alter metabolism, and promote survival signaling [13,14]. Furthermore, the stroma establishes an immunosuppressive niche through modified ECM composition and cytokine profiles, facilitating tumor immune evasion [11,15,16,17]. These biomechanical and biochemical interactions can induce drug resistance by activating survival pathways and upregulating drug efflux mechanisms [18,19,20,21,22].

Patient-derived organoids (PDOs) have emerged as promising tools for studying these complex tumor–stroma interactions [10,23,24]. However, conventional PDO models often fail to recapitulate the mechanical properties of the dense PDAC stroma, limiting their predictive value [24,25]. To address this gap, we developed a biomimetic platform using transglutaminase crosslinked collagen matrices with tunable density and stiffness [26,27]. This approach is particularly relevant, as transglutaminase 2 (TG2) overexpression in PDAC correlates with increased ECM stiffness, invasiveness, and chemoresistance [28,29].

In this study, we leverage this mechanically tunable organoid platform to investigate how matrix stiffness influences the efficacy of gemcitabine and its lipophilic derivative, 4-N-stearoyl gemcitabine (Gem-S). By examining the interplay between mechanical forces, drug response, and resistance mechanisms, we aim to inform both drug design and therapeutic strategies for PDAC treatment.

## 2. Materials and Methods

### 2.1. Materials and Matrix Development

Unless otherwise stated, all chemicals and reagents were purchased from Sigma-Aldrich (St. Louis, MO, USA). Details on the preparation of microbial transglutaminase (Tg) and gelatin gel were described previously [30,31,32]. Briefly, gelatin Type A 300 Bloom (Sigma Aldrich, St. Louis, MO, USA) was dissolved and made into final concentrations of 3%, 6%, and 9%. Tg from Streptomyces mobaraense was obtained from Ajinomoto (Tokyo, Japan) and was further purified with SP Sepharose Fast Flow beads (Sigma Aldrich, St. Louis, MO, USA). The activity of Tg was titrated by the o-phthaldialdehyde (OPA) assay using casein as a substrate, and the protein concentration was tested by the Bradford method (Bio-Rad, Hercules, CA, USA) utilizing bovine serum albumin (BSA) as a standard [33]. Gemcitabine (Gem) was obtained from AK Scientific, while Gem-stearyl was synthesized according to previously established protocols [34]. All compounds were prepared as 10 mmol/L DMSO stock solutions for in vitro studies. Rescue of the cell viability was conducted using 1 mM N-Acetyl-L-cysteine (Sigma Aldrich, St. Louis, MO, USA).

### 2.2. Matrix Characterization

Matrix mechanical properties were characterized using a novel non-contact ultrasound technique [35]. Three ultrasound transducers positioned 5 mm above the matrix surface measured the rheological properties. A high-power ultrasonic transducer (440 kPa shear stress) with an oblique angle provided mechanical input, while the reflected acoustic energy was captured by secondary transducers. Deformation was measured with 1 µm spatial resolution, enabling precise stress–strain characterization over time.

### 2.3. Patient-Derived Cell Culture

Seven patient-derived pancreatic ductal adenocarcinoma (PDAC) cells were generated from surgical sectioned pancreatic adenocarcinoma in the laboratory of the University of Florida (under IRB protocol 201600873) as describe previously [36]. These cells were expanded in a growth medium of high-glucose Dulbecco’s modified Eagle’s medium (DMEM, Genesee Scientific, El Cajon, CA, USA) supplemented with 10% (*v*/*v*) fetal bovine serum (FBS; Sigma, St. Louis, MO, USA) and 1% (*v*/*v*) penicillin–streptomycin (PS, Genesee Scientific, El Cajon, CA, USA) in a humidified atmosphere of 5% CO_2_ at 37 °C. The medium was changed every 2–3 days. When the cells reached 70–80% confluence, they were harvested using 0.05% trypsin-EDTA solution (Gibco, Thermo Fisher Scientific, Waltham, MA, USA). Cultures were confirmed for mycoplasma negativity using the MycoAlert Mycoplasma Detection Kit (catalog No. LT07–318, Lonza, Cambridge, MA, USA).

### 2.4. Three-Dimensional Organoid Culture and Drug Treatment

Organoids were generated by embedding PDAC cells at a concentration of 2 × 10^6^ cells/mL in gelatin–Tg matrices. Cell suspensions were mixed with sterile gelatin solution (6% Type A and 6% Type B) and purified Tg. Twenty microliters of cell matrix mixture were cast per well in 48-well suspension plates and allowed to solidify at 37 °C for 45 min before adding 500 μL culture medium. Organoids were cultured for 7 days prior to drug treatment, with medium changes every 3 days.

### 2.5. Cell Viability and Proliferation Analysis

Cell viability was assessed using multiple complementary methods. For 2D cultures, viability was determined using the MTT assay, with absorbance measured at 560 nm. In 3D cultures, viability was evaluated using both the CellTiter-Glo^®^3D assay and CCK-8 assay (450 nm). Additionally, cellular viability was visualized using the LIVE/DEAD Viability/Cytotoxicity Kit (Thermo Fisher Scientific) following the manufacturer’s protocol. For proliferation assessment, cells were counted directly in 2D cultures, while 3D cultures required initial collagenase digestion (20 units Type II collagenase, 4 h) to release cells prior to counting. All measurements were performed in triplicate and normalized to appropriate controls. Cell counting was performed to determine cell growth and doubling times. In 2D culture, the monolayer cells were harvested using 0.05% trypsin-EDTA at 3 days after seeding. In 3D culture, the doubling times were deduced from two time points (6 and 12 days after seeding). The Col-T gel cell constructs were washed twice with PBS and digested with 20 units of Type II microbial collagenase (Worthington, NJ, USA) for 4 h to release cells. All the cells were collected and counted with a cell counter (Beckman Coulter, Brea, CA, USA).

### 2.6. Drug Response Studies

Dose–response experiments utilized a 7-point dilution series (10^−2^ to 10^−8^ M or 10^−4^ to 10^−10^ M) in both 2D and 3D formats. For 3D studies, organoids were established for 7 days before drug exposure for 3 or 6 days. The results were normalized to the vehicle controls. Cell viability was assessed using the LIVE/DEAD™ Cell Imaging Kit (Invitrogen Cat# R37601, Carlsbad, CA, USA) according to the manufacturer’s instructions, with modification. Briefly, 3D cells were washed with PBS and incubated with a working solution containing 2 μM calcein AM and 4 μM ethidium homodimer-1 in PBS for 60 min in the dark followed by fluorescence imaging (EVOS, Thermo Fisher Scientific, MA, USA).

### 2.7. IHC Staining

For immunohistochemical analysis, the Col-T gel cell construct was fixed in 10% neutral formalin for 10 min and incubated in peroxidase suppressor solution (Thermo Fisher Scientific, Pittsburg, PA, USA) for 30 min and then in the blocking buffer (5% BSA in the mixture of Tris-buffered saline and Tween-20) for 30 min. The construct was incubated at 4 °C overnight with the primary antibody E-cadherin, Ki67, HIF, and vimentin (dilution = 1:400, cat. PA5-32178, Thermo Fisher Scientific, Pittsburgh, PA, USA), then with FITC-conjugated secondary antibody (dilution = 1:1000, cat. 65-111, Thermo Fisher Scientific) for 30 min and DAPI dihydrochloride (dilution = 1:100,000, cat. 40011, Biotium Inc., Fremont, CA, USA) (nuclei staining) in the dark for 5 min. After stopping the reaction by washing with TBST, cell images were acquired by an EVOS fluorescence microscope. Cell morphology and cytoskeleton organization were visualized by F-actin staining. Positive cell counts were analyzed using ImageJ-assisted (https://imagej.net/ij/) counting methodology. Values represent the total number of cells counted from one representative section per condition.

### 2.8. Transcriptional Analysis

Total RNA was extracted from different culture conditions using the RNeasy Mini Kit (Qiagen, Thousand Oaks, CA, USA). RNA sequencing libraries were prepared using the SMART-Seq Stranded kit (Takara, Kusatsu, Japan) and sequenced on the Illumina NovaSeq 6000 platform with 100 bp paired-end reads, achieving >20 million reads per sample. Raw sequencing data underwent quality control and processing through a systematic bioinformatics pipeline. Initial adapter sequences were removed using Trim Galore (Version 0.6.5), followed by alignment of the trimmed reads to the GRCh38 reference genome using STAR (version 2.7.10a). Gene-level quantification was performed using RSEM (version 1.3.1), and the resulting count data were normalized using TMM normalization in edgeR. Differential expression analysis was conducted using the edgeR package (3.34.0) in R. For the validation of key genes, RT-qPCR was performed using the iTaq™ Universal SYBR^®^ Green System (Bio-Rad, Hercules, CA, USA), with ACTB serving as the internal control. All samples passed quality control based on the manufacturer’s standards, and expression changes were calculated relative to the control conditions. Primers used in the study are listed in the following table (Table 1).

### 2.9. Statistical Analysis

Group comparisons were performed using ANOVA with Tukey’s post hoc test or two-sided unpaired Student’s *t*-test as appropriate. Significance was set at *p* < 0.05.

## 3. Results

### 3.1. Distinct Matrix Mechanics Dictate PDAC Organoid Architecture and Growth

**Figure 1:**
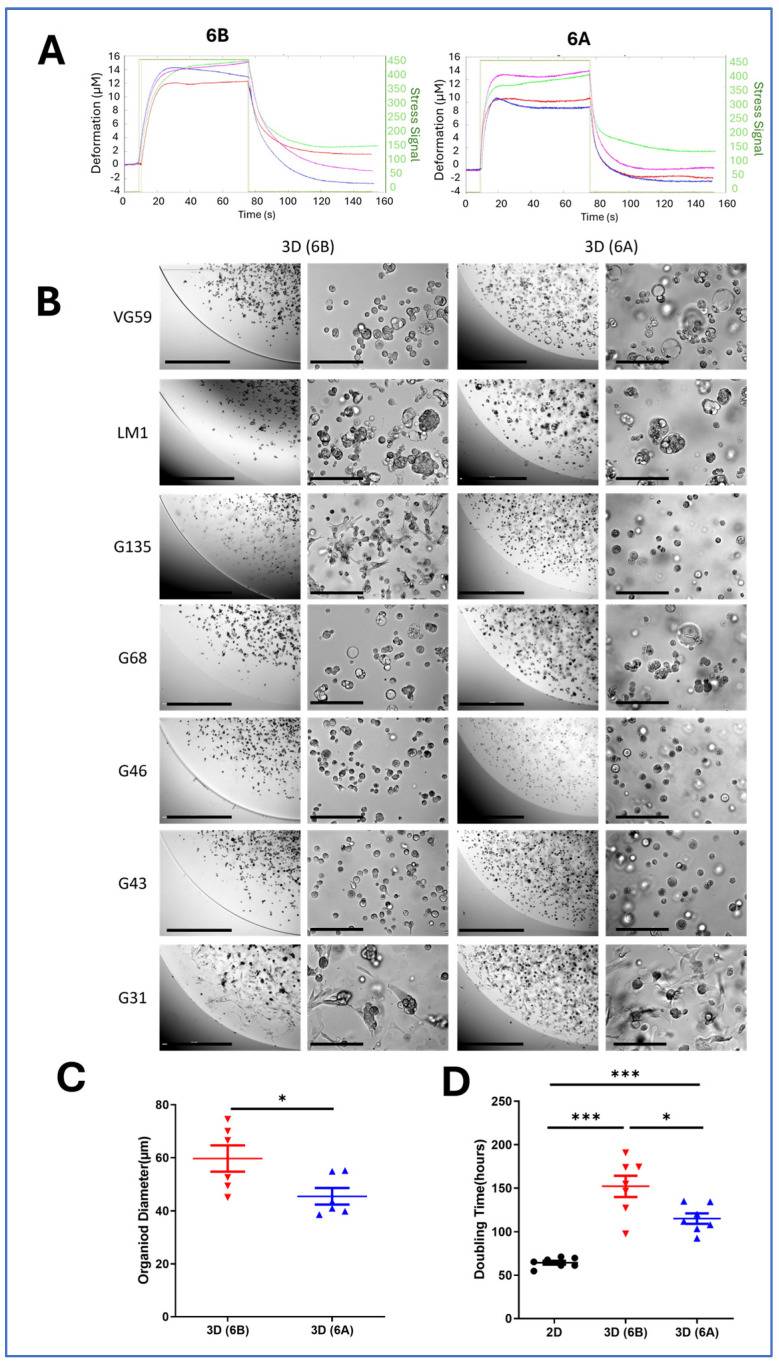
Matrix mechanics govern PDAC organoid architecture and growth dynamics. (**A**) Non-invasive rheological characterization of cell-embedded collagen-transglutaminase (Col-Tgel) matrices. Ultrasound transducers measured matrix deformation under 440 kPa shear stress over time. Representative stress–strain curves demonstrate distinct viscoelastic properties between matrix formulations. (**B**) Representative phase contrast micrographs of seven patient-derived PDAC organoids cultured in stiff (6A) vs. soft (6B) matrices for 6–8 days. Semi-dome 3D configuration enabled the visualization of matrix-dependent morphological adaptations. Scale bars: 1000 µm (low magnification) and 100 µm (high magnification). (**C**) Quantitative analysis of organoid size using ImageJ software (version 1.5.4). Measurements represent organoid diameters from G68-derived cells after 8 days of culture in matrices 6A and 6B. Data presented as mean ± SD; * *p* < 0.05, *** *p* < 0.001 (Student’s *t*-test). (**D**) Comparative analysis of cell proliferation kinetics across culture conditions. Doubling times were calculated following collagenase-mediated matrix digestion and automated cell counting (Beckman Coulter). Data shown as mean ± SD from three independent experiments.

We developed two distinct viscoelastic matrices using 6% gelatin with differential transglutaminase crosslinking rates, achieved through acidic (6A) vs. alkaline (6B) processing. Creep experiments under 440 kPa stress revealed distinct mechanical signatures: the highly crosslinked 6A matrix exhibited minimal deformation (6–8 µm) with rapid recovery (20 s), while the less crosslinked 6B matrix showed greater deformation (12–15 µm) and slower recovery (40 s) (Figure 1A). This approach enabled us to study cellular responses across different mechanical environments while maintaining a consistent matrix composition.

Within these defined matrices, seven patient-derived PDAC cell lines successfully formed organoids over 6–8 days, displaying both patient-specific and matrix-dependent morphological characteristics. The organoids exhibited diverse architectures, ranging from compact spheroids to loose cell clusters. These morphological differences were observed both across different patient-derived PDAC lines and within the individual lines, suggesting that matrix mechanics play a role in shaping organoid architecture beyond intrinsic cell line properties (Figure 1B). The semi-dome 3D culture configuration facilitated direct visualization of these matrix-dependent morphological adaptations, allowing us to capture distinct organoid growth patterns in response to differential matrix mechanics (Figure 1B). Quantitative analysis revealed that matrix mechanics significantly influenced organoid size: cells consistently formed larger structures in the softer 6B matrix compared to the stiffer 6A matrix (59.69 µm vs. 45.49 µm, *p* < 0.01) (Figure 1C).

Matrix stiffness profoundly impacted cellular proliferation rates, with growth kinetics analysis revealing distinct patterns across culture conditions. Cells in the 2D monolayer showed the fastest growth with a doubling time of approximately 64.4 h, while those in the 3D soft matrix (6B) exhibited intermediate growth rates with doubling times around 115.2 h. The slowest proliferation was observed in the stiff 3D matrix (6A), where the doubling times extended to approximately 152.2 h. This progressive slowdown in proliferation from 2D to soft 3D to stiff 3D environments suggests that increasing the matrix stiffness creates a more restrictive growth environment, which more closely mimics the challenges tumor cells face in vivo, where a dense, stiff extracellular matrix can limit proliferation while promoting other tumor behaviors such as invasion and metastasis (Figure 1D).

### 3.2. Matrix Mechanics Drive Distinct Transcriptional Programs in PDAC Organoids

**Figure 2:**
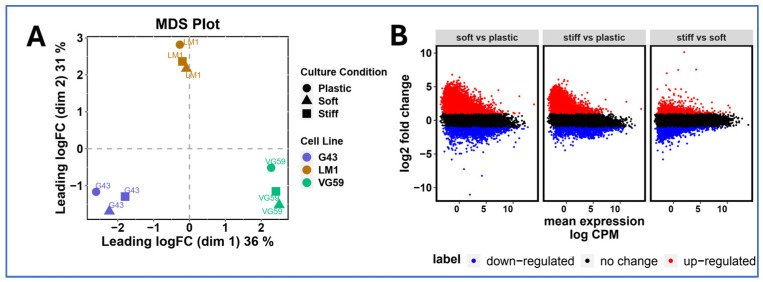
Matrix-dependent transcriptional reprogramming in PDAC organoids. (**A**) Multidimensional scaling (MDS) plot of RNA sequencing data from three patient-derived PDAC lines (G43, VG59, and LM-1) cultured under different conditions (2D monolayer, soft matrix 6B, and stiff matrix 6A). Analysis based on the top 1000 most variable genes demonstrates distinct transcriptional clustering by culture condition. Each point represents an individual sample, with distances reflecting relative transcriptional similarities. (**B**) Mean difference plots comparing gene expression between culture conditions. Each point represents an individual gene, with red dots indicating upregulated genes (fold change > 2) and blue dots indicating downregulated genes (fold change < −2) relative to the 2D culture. Gray dots represent genes with less than two-fold change in expression. Results shown for three pairwise comparisons: 3D soft matrix (6B) vs. 2D culture, 3D stiff matrix (6A) vs. 2D culture, and 3D stiff matrix (6A) vs. 3D soft matrix (6B). RNA sequencing performed with >20 million reads per sample. Data analyzed using edgeR following TMM normalization. *n* = 3 biological replicates per condition.

To elucidate how matrix mechanics influence the cellular phenotype, we performed comprehensive transcriptional profiling of three patient-derived organoid lines (G43, VG59, and LM-1) across different culture conditions. Multidimensional scaling (MDS) analysis of the top 1000 most variable genes revealed distinct transcriptional signatures that were segregated based on the culture conditions (2D, soft matrix 6B, and stiff matrix 6A) (Figure 2A). This clear separation suggests that both three-dimensionality and matrix stiffness fundamentally reshape gene expression programs.

Differential expression analysis highlighted substantial transcriptional reprogramming between the culture conditions. Mean difference plots revealed widespread gene expression changes, with numerous genes showing greater than two-fold changes (red: upregulated, FC > 2; blue: downregulated, FC < −2) in 3D cultures compared to conventional 2D culture (Figure 2B). This global shift in gene expression patterns suggests that the mechanical microenvironment profoundly influences the cellular state beyond simple morphological adaptations.

### 3.3. Matrix Mechanics Modulate Drug Sensitivity and Survival Pathways in PDAC Organoids

**Figure 3:**
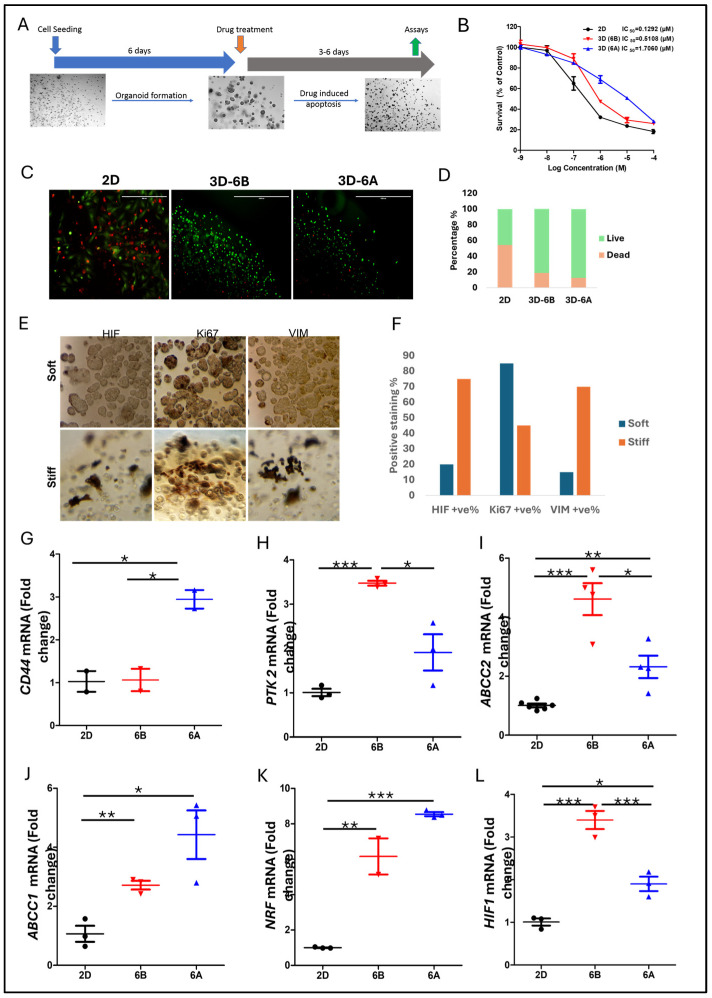
Matrix stiffness orchestrates drug resistance programs in PDAC patient-derived organoids. (**A**) Schematic representation of the experimental design. PDAC PDOs were established in soft (6B) and stiff (6A) matrices for 6 days prior to gemcitabine (Gem) treatment. Cell viability was quantified via the CellTiterGlo luminescence assay following 72 h drug exposure. (**B**) Dose–response curves for G43-derived organoids treated with gemcitabine. The IC_50_ values demonstrate progressive resistance from the 2D monolayer to soft and stiff 3D cultures (4–12-fold increase in IC_50_). Data presented as mean ± SD from three independent experiments. (**C**,**D**) Quantitative assessment of cell death using the Live/Dead fluorescence assay following 1 µM gemcitabine treatment. Representative images and quantification showing reduced apoptosis in 3D cultures compared to 2D conditions. Scale bar = 100 µm. (**E**) Immunochemical analysis of key cellular markers across culture conditions. Representative images showing the differential expression of HIF-1α (hypoxia), Ki67 (proliferation), and vimentin (EMT) in 2D, soft 3D, and stiff 3D environments. Scale bar = 100 µm. (**F**) Quantification of representative IHC images; the values represent the total number of cells counted from one representative section per condition. (**G**–**L**) RT-qPCR analysis of mechanosensitive gene expression in G68-derived organoids. Data normalized to the 2D culture and ACTB expression, showing matrix-dependent regulation of stemness regulators CD44 (**G**) and PTK2 (**H**), drug efflux transporters ABCC2 (**I**) and ABCC1 (**J**), and stress response factors NRF2 (**K**) and HIF-1α (**L**). Data represent mean ± SEM from three biological replicates. * *p* < 0.05, ** *p* < 0.01, and *** *p* < 0.001 by one-way ANOVA with Tukey’s post hoc test.

We systematically evaluated how matrix mechanics influence chemoresistance using established PDAC organoids in matrices of distinct stiffness (Figure 3A). The initial studies with G43-derived organoids revealed substantially reduced gemcitabine (Gem) sensitivity in 3D cultures compared to 2D monolayers, with further resistance developing in stiffer matrices (Figure 3B). Quantitative analysis of the IC_50_ values demonstrated that organoids required 4–12-fold higher drug concentrations for equivalent growth inhibition compared to the monolayer cultures, though the magnitude varied among patient samples. Live/dead staining confirmed these findings, showing significantly reduced apoptosis in 3D cultures compared to 2D conditions at 1 µM Gem, as quantified in Figure 3C,D.

Immunofluorescence analysis (Figure 3E,F) revealed matrix stiffness-dependent changes in key cellular markers, where stiffer matrices exhibited markedly increased HIF-1α and vimentin staining intensity compared to the soft matrices. In contrast, Ki67 positivity was notably reduced in stiff conditions, particularly in organoid cores, suggesting the differential regulation of proliferation based on matrix mechanics. The enhanced vimentin expression in stiff matrices points to the potential activation of EMT-like phenotypes.

Mechanistic investigation through transcriptional profiling revealed several stiffness-regulated pathways contributing to chemoresistance. Analysis of the stemness markers showed enhanced CD44 expression specifically in stiff matrices, while PTK2 expression increased in both 3D conditions, suggesting the matrix-dependent regulation of stem cell-like properties (Figure 3G,H). The drug efflux machinery also showed significant adaptation, with elevated expression of multidrug transporters ABCC1 and ABCC2 in 3D cultures, though their expression patterns varied with the matrix stiffness (Figure 3I,J).

Furthermore, we observed the significant activation of stress response pathways in matrix-specific organoids. The NRF2 pathway showed enhanced activity, indicating an elevated antioxidant response. Concurrent with this, we noted increased HIF-1 signaling, likely resulting from restricted oxygen diffusion and metabolic adaptations within the 3D environment (Figure 3I,J). These findings suggest that matrix mechanics orchestrate a complex adaptive response involving stemness maintenance, drug efflux, and stress response pathways, collectively contributing to chemoresistance.

### 3.4. Stearoyl-Modified Gemcitabine Shows Enhanced Efficacy in Stiff Matrices

**Figure 4:**
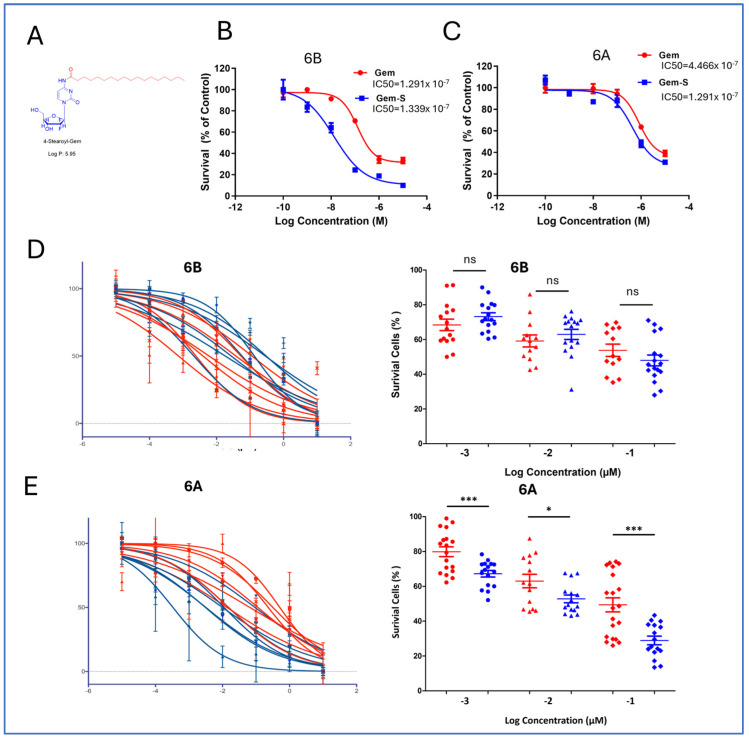
Matrix stiffness differentially modulates the efficacy of stearoyl-modified gemcitabine in PDAC organoids. (**A**) Chemical structure and schematic representation of gemcitabine modification, showing stearoyl conjugation at the 4N position to generate Gem-S. (**B**,**C**) Comparative drug response analysis in G68 cells across matrix conditions. Dose–response curves demonstrate differential sensitivity to Gem and Gem-S in soft (6B) vs. stiff (6A) matrices. The IC_50_ values reveal significantly enhanced Gem-S potency in stiff matrices (1.291 × 10^−7^ M vs. 1.339 × 10^−8^ M, *p* < 0.001). Data are presented as mean ± SD from three independent experiments. (**D**,**E**) Matrix-dependent drug sensitivity across seven patient-derived organoid lines. Cell viability assessed after 72 h drug exposure in soft (6B) and stiff (6A) matrices. Similar drug responses were observed in soft matrices, while stiff matrices show enhanced sensitivity to Gem-S compared to unmodified gemcitabine. Data were normalized to the vehicle controls and presented as mean ± SEM. Statistical significance was determined by two-sided unpaired Student’s *t*-test (ns *p* > 0.05, * *p* < 0.01, and *** *p* < 0.0001).

To combat matrix-induced chemoresistance, we evaluated a modified version of gemcitabine featuring stearoyl conjugation at the 4N position (Gem-S) (Figure 4A). The initial studies using G68 cells revealed a striking matrix-dependent drug response pattern. While both compounds showed similar efficacy in the 2D cultures, Gem-S demonstrated significantly enhanced potency in stiff matrices compared to soft matrices (IC_50_: 1.291 × 10^−7^ M vs. 1.339 × 10^−8^ M, *p* < 0.001) (Figure 4B).

We extended this analysis to PDOs derived from seven patients to validate these findings across a broader biological context. In soft matrices (6B), Gem and Gem-S exhibited comparable inhibitory effects, with no significant differences in therapeutic response. However, in stiff matrices (6A), a clear differentiation emerged: PDOs showed markedly higher sensitivity to Gem-S compared to unmodified gemcitabine (Figure 4D,E). This matrix-dependent divergence in drug efficacy suggests that the stearoyl modification enables Gem-S to overcome specific resistance mechanisms that emerge in mechanically restrictive environments.

### 3.5. Gem-S Efficacy Is Mediated Through Oxidative Stress in Stiff Matrix Environments

**Figure 5:**
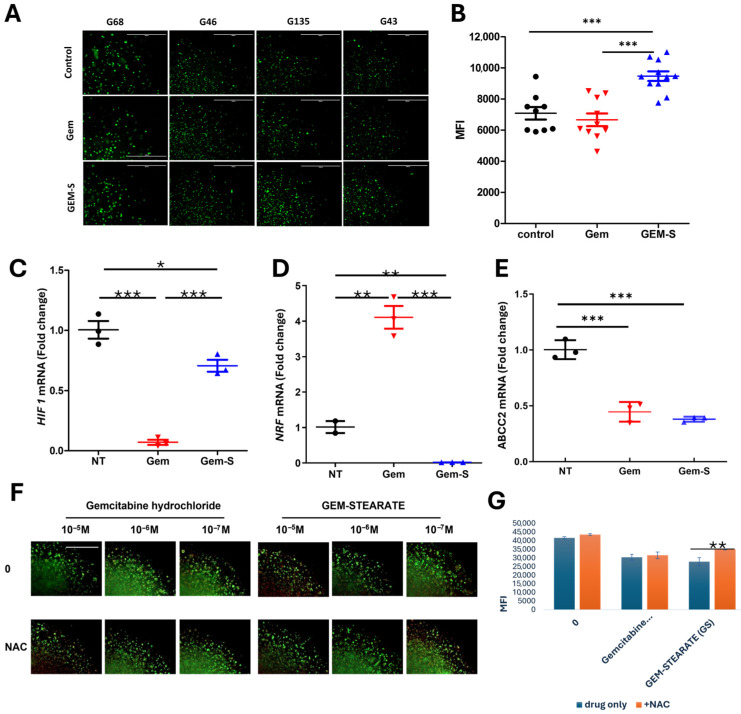
Oxidative stress mediates enhanced Gem-S efficacy in stiff matrix environments. (**A**) Visualization of reactive oxygen species (ROS) in PDOs cultured in stiff (6A) matrices following 48 h drug exposure. Representative MitoROX fluorescence images from four independent PDO lines captured across quarter-sections of 3D droplets. Scale bar = 1000 µm. (**B**) Quantitative analysis of the ROS levels. ImageJ (version 1.5.4)-based fluorescence intensity measurements demonstrating significantly elevated ROS production in Gem-S-treated organoids compared to Gem treatment (mean ± SD, *** *p* < 0.0001, Student’s *t*-test). (**C**–**E**) Differential regulation of stress response pathways and drug resistance mechanisms. RT-qPCR analysis showing drug-specific effects on HIF-1α expression (hypoxic response), NRF2 expression (antioxidant defense), and ABCC2 expression (drug efflux). Data were normalized to the vehicle control and presented as mean ± SEM from three independent experiments. (**F**,**G**) Antioxidant rescue experiments. (**F**) Representative Live/Dead fluorescence images following Gem-S treatment with or without N-acetylcysteine (NAC) co-treatment. (**G**) Quantification of the mean fluorescence intensity (MFI) demonstrating the partial rescue of cell viability by NAC in Gem-S-treated organoids. Data are presented as mean ± SD, * *p* < 0.05 and ** *p* < 0.01 by one-way ANOVA with Tukey’s post hoc test.

To elucidate the molecular mechanisms underlying differential drug responses in stiff matrices, we performed comprehensive marker analysis following treatment. Gem-S treatment induced significantly higher levels of reactive oxygen species (ROS) compared to unmodified gemcitabine in the 6A matrices (Figure 5A,B), suggesting a distinct mode of action involving oxidative stress.

A key mechanistic insight emerged from the analysis of stress response pathways. While gemcitabine treatment activated NRF2, a master regulator of antioxidant responses, Gem-S notably suppressed NRF2 expression (Figure 5C). This differential regulation of the antioxidant defense system suggests that Gem-S may achieve enhanced efficacy by compromising the cellular stress responses. Concurrent with NRF2 suppression, Gem-S treatment elevated HIF-1 expression (Figure 5D), indicating a complex interplay between oxidative stress and hypoxic responses.

Surprisingly, multidrug transporter expression remained unchanged or decreased following Gem-S treatment (Figure 5E), suggesting that enhanced drug efficacy operates independently of classical drug efflux mechanisms. To validate the role of oxidative stress in Gem-S-mediated cell death, we performed rescue experiments using the antioxidant N-acetylcysteine (NAC). NAC treatment partially reversed Gem-S cytotoxicity (Figure 5F,G), confirming that redox modulation is a key component of Gem-S’s therapeutic mechanism in stiff matrix environments.

Figure 5F,G effect of N-acetylcysteine (NAC) co-treatment: Live/Dead staining images after co-treatment with NAC and mean fluorescence intensity (MFI) plots generated from the images, showing that NAC partially rescued cell viability after Gem-S treatment.

## 4. Discussion

Our study reveals a novel mechanistic insight into how matrix stiffness influences chemotherapeutic efficacy in pancreatic cancer, demonstrating that stearoyl-modified gemcitabine (Gem-S) exhibits enhanced potency, specifically in stiff environments characteristic of advanced PDAC. This finding is particularly significant given that conventional gemcitabine’s efficacy often diminishes as tumors progress and become more fibrotic, suggesting that Gem-S could be especially valuable for treating late-stage disease.

To investigate matrix-dependent drug responses, we developed a tunable organoid platform that better reflects the mechanical complexity of PDAC. While most patient-derived organoids (PDOs) are cultured in soft Matrigel [23,24,25], our gelatin-based system enables precise control over matrix stiffness while maintaining a consistent biochemical composition [26,32]. By varying crosslinking degrees through distinct gelatin hydrolysis methods (acid vs. alkaline), we created environments that more accurately mirror the progressive stiffening observed in PDAC development [36]. This approach revealed that PDAC cells demonstrate remarkable plasticity in response to mechanical cues, adapting their morphology, growth patterns, and drug sensitivity in a patient-specific manner [37,38].

The mechanical properties of the matrix profoundly influenced the cellular phenotype and drug response [39]. In stiffer matrices, PDAC cells exhibited enhanced stemness features, adopted more compact morphologies, and showed increased resistance to conventional gemcitabine [39]. This resistance correlated with the upregulation of ABC transporters and activation of stress response pathways, particularly in hypoxic regions [40,41]. Importantly, these adaptations varied among the patient samples, highlighting the need for personalized therapeutic approaches that consider both mechanical and molecular factors.

Gemcitabine remains a cornerstone therapy for PDAC, but its efficacy is often limited by the development of resistance. To address this, we investigated 4-N-stearoyl gemcitabine (Gem-S), a lipophilic fatty acid derivative of gemcitabine that has shown improved drug delivery and efficacy in other studies [34,42,43]. The superior efficacy of Gem-S in stiff matrices appears to stem from a unique dual mechanism. While conventional gemcitabine activated the Nrf2-mediated antioxidant response [44], Gem-S suppressed this protective pathway while simultaneously increasing reactive oxygen species (ROS) production. This creates a vulnerability specifically in mechanically stressed cells, which typically rely heavily on antioxidant defenses to manage elevated baseline stress levels [45]. The concurrent reduction in HIF expression further compromises cellular adaptation to the challenging microenvironment, potentially explaining why Gem-S shows particular efficacy in stiff matrices where these stress responses are critical for survival.

The relationship between mechanical and oxidative stress emerges as a key therapeutic opportunity. Previous studies have shown that stiff matrices increase baseline cellular stress through mechanotransduction pathways [46,47,48]. Our findings suggest that Gem-S exploits this elevated stress state by simultaneously increasing ROS production and compromising antioxidant defenses. This may be particularly effective against cancer stem cells, which typically maintain careful redox balance to preserve their stemness. The stearoyl modification may also enhance drug accumulation in mechanically stressed cells through altered membrane properties or lipid metabolism [49], though this requires further investigation.

These findings have important clinical implications. First, they suggest that tumor stiffness could serve as a biomarker for predicting Gem-S efficacy, potentially allowing better patient stratification. Second, the identified mechanism suggests promising combination strategies, such as pairing Gem-S with inhibitors of antioxidant pathways or mechanical signaling. Finally, our results highlight the importance of considering matrix mechanics in drug development and testing, as conventional soft culture systems may fail to predict efficacy in the stiff PDAC environment.

Several questions warrant further investigation. The molecular mechanisms linking matrix stiffness to Nrf2 regulation and ROS generation need detailed elaboration. The role of lipid metabolism in Gem-S’s matrix-dependent effects requires clarification. Additionally, the potential for resistance development and optimal timing of Gem-S administration in the disease course should be examined.

## 5. Conclusions

In conclusion, our study not only identifies Gem-S as a promising agent for treating mechanically rigid PDAC but also establishes a new paradigm for considering matrix mechanics in drug development. By demonstrating how mechanical and oxidative stress pathways intersect to influence drug efficacy, this work opens up new avenues for therapeutic intervention in PDAC and potentially other stiff tumors.

## Figures and Tables

**Table 1:** PCR primers.

Primer	Sequence
ABCC1 F	CCGTGTACTCCAACGCTGACAT
ABCC1 R	ATGCTGTGCGTGACCAAGATCC
ABCC2 F	GCCAACTTGTGGCTGTGATAGG
ABCC2 R	ATCCAGGACTGCTGTGGGACAT
NRF-2 F	AAATTGAGATTGATGGAACAGAGAA
NRF-2 R	TATGGCCTGGCTTACACATTCA
HIF 1 F	TATGAGCCAGAAGAACTTTTAGGC
HIF 1 R	CACCTCTTTTGGCAAGCATCCTG
PTK 2 F	GCCTTATGACGAAATGCTGGGC
PTK 2 R	CCTGTCTTCTGGACTCCATCCT
CD44 F	CCAATGCCTTTGATGGACCA
CD44 R	TGTGAGTGTCCATCTGATTC

## Data Availability

Data are available upon request.
